# Long Non-Coding RNAs Responsive to Salt and Boron Stress in the Hyper-Arid Lluteño Maize from Atacama Desert

**DOI:** 10.3390/genes9030170

**Published:** 2018-03-20

**Authors:** Wilson Huanca-Mamani, Raúl Arias-Carrasco, Steffany Cárdenas-Ninasivincha, Marcelo Rojas-Herrera, Gonzalo Sepúlveda-Hermosilla, José Carlos Caris-Maldonado, Elizabeth Bastías, Vinicius Maracaja-Coutinho

**Affiliations:** 1 Departamento de Producción Agrícola, Facultad de Ciencias Agronómicas, Universidad de Tarapacá, Arica 1000000, Chile; sfcninasivincha@gmail.com (S.C.-N.); carlos.caris.m@gmail.com (J.C.C.-M.); ebastias@uta.cl (E.B.); 2 Centro de Genómica y Bioinformática, Facultad de Ciencias, Universidad Mayor, Santiago 8580745, Chile; raul.arias.carrasco@gmail.com (R.A.-C.); marcelo.rojas.herrera@gmail.com (M.R.-H.); gosepulvedah@gmail.com (G.S.-H.); 3 Departamento de Bioquímica y Biologia Molecular, Facultad de Ciencias Químicas y Farmacéuticas, Universidad de Chile, Santiago 8380494, Chile; 4 Instituto Vandique, João Pessoa 58000-000, Brazil; 5 Beagle Bioinformatics, Santiago 7500554, Chile

**Keywords:** long non-coding RNAs, lncRNAs, salt tolerance, boron tolerance, Lluta valley, Lluteño, *Zea mays*, *trans*-NAT

## Abstract

Long non-coding RNAs (lncRNAs) have been defined as transcripts longer than 200 nucleotides, which lack significant protein coding potential and possess critical roles in diverse cellular processes. Long non-coding RNAs have recently been functionally characterized in plant stress–response mechanisms. In the present study, we perform a comprehensive identification of lncRNAs in response to combined stress induced by salinity and excess of boron in the Lluteño maize, a tolerant maize landrace from Atacama Desert, Chile. We use deep RNA sequencing to identify a set of 48,345 different lncRNAs, of which 28,012 (58.1%) are conserved with other maize (B73, Mo17 or Palomero), with the remaining 41.9% belonging to potentially Lluteño exclusive lncRNA transcripts. According to B73 maize reference genome sequence, most Lluteño lncRNAs correspond to intergenic transcripts. Interestingly, Lluteño lncRNAs presents an unusual overall higher expression compared to protein coding genes under exposure to stressed conditions. In total, we identified 1710 putatively responsive to the combined stressed conditions of salt and boron exposure. We also identified a set of 848 stress responsive potential *trans* natural antisense transcripts (*trans*-NAT) lncRNAs, which seems to be regulating genes associated with regulation of transcription, response to stress, response to abiotic stimulus and participating of the nicotianamine metabolic process. Reverse transcription-quantitative PCR (RT-qPCR) experiments were performed in a subset of lncRNAs, validating their existence and expression patterns. Our results suggest that a diverse set of maize lncRNAs from leaves and roots is responsive to combined salt and boron stress, being the first effort to identify lncRNAs from a maize landrace adapted to extreme conditions such as the Atacama Desert. The information generated is a starting point to understand the genomic adaptabilities suffered by this maize to surpass this extremely stressed environment.

## 1. Introduction

Comprehensive genome-wide transcriptional studies revealed that a large fraction of the eukaryotic genome is transcribed, with only about 2% of them translated into proteins [1]. Most of these transcripts conform a heterogeneous population of non-coding RNAs (ncRNAs) [2]. The spatial–temporal expression, varied subcellular localization, tissue and stage specificity reinforces its crucial role as central components of an extensive eukaryotic RNA controlled network [1,2,3]. In general, ncRNAs are classified into different families according to their length, genomic location, biological function and sequence/structure conservation [4,5]. They also can be classified as unspliced (mono-exonic) or spliced (multi-exonic), i.e., possess more than one exon [6]. Non-coding transcripts can be grouped as housekeeping or regulatory ncRNAs. Housekeeping ncRNAs are usually expressed constitutively and include ribosomal, transfer, small nuclear and small nucleolar RNAs. Based on their size, regulatory ncRNAs shorter than 200 nucleotides are usually classified as small/short ncRNAs, while those longer than 200 nucleotides are classified as long non-coding RNAs (lncRNAs) [7,8].

The last two decades has seen significant progress on the understanding of the functional roles and molecular mechanisms of small RNAs in plants. Well-studied classes include microRNAs (miRNAs), small interfering RNAs (siRNAs) and natural antisense siRNAs (nat-siRNAs), which possess essential roles on transcriptional and post-transcriptional regulation of gene expression [9,10]. Nonetheless, the regulatory roles of lncRNAs are only beginning to be recognized, and the molecular basis of lncRNA-mediated gene regulation is still poorly understood [11]. The advent of high-throughput expression profiling technologies resulted in the identification of thousands of lncRNAs in model plants, such as *Arabidopsis thaliana* [12,13], *Medicago truncatula* [14], *Triticum aestivum* [15], *Oryza sativa* [16], *Solanum lycopersicum* [10] and *Zea mays* [17]. There is growing evidence indicating that plant lncRNAs have critical roles in cell differentiation, epigenetic modification, genomic imprinting and stress tolerance [18,19,20]. These data reinforce the fact that lncRNAs are functionally relevant, rather than remnants of transcriptional processes, as initially proposed [21].

Emerging evidence suggests that some mechanisms regulated by lncRNAs are conserved between plants and animals [22]. Plant long non-coding transcripts have been less studied and identified, with only a few functionally characterized. In *A. thaliana*, some interesting examples are the Cold Assisted Intronic Non-coding RNA (COLDAIR); the Cold Induced Long Antisense Intergenic RNA (COOLAIR); the Alternative Splicing Competitor RNA (ASCO-lncRNA); and the Induced by Phosphate Starvation 1 (IPSI1) lncRNA. COLDAIR is transcribed from the intron 1 of the Flowering Locus C (*FLC*) gene, and participates in *FLC* repression by vernalization, through chromatin modifications performed by the recruitment of Polycomb Repressive Complex 2 (PRC2), by directly interacting with Curly Leaf (*CLF*) [23]. COOLAIR is an alternatively spliced natural antisense transcript (NAT), transcribed from the antisense orientation of *FLC* gene, which has an important role in modulating *FLC* expression during vernalization [24,25]. ASCO-lncRNA interacts with Nuclear Speckle RNA Binding Protein (NSR) to modulate the alternative splicing activity of several NSR-regulated messenger RNAs (mRNAs) in auxin signaling pathway [26]. IPSI1 lncRNA contains a motif with sequence complementarity to the phosphate (P_i_) starvation-induced miRNA miR-399, which directly represses the expression of *PHO2* gene by mRNA cleavage. IPSI1 might work as miRNAs target mimics, which exert its function by binding miRNAs in a target mimicry mechanism to sequestrate and inhibit the miRNA regulatory roles on their target coding genes [27]. In *M. truncatula*, Early Nodulin 40 (ENOD40) lncRNA is required for the root nodules organogenesis and is highly conserved in legumes, rice and maize [28]. The ENOD40 transcript interacts directly with *M. trunculata* Nuclear Speckles RNA-binding protein 1 (mtNSR1) to induce NSR1 protein re-localization from nuclear to cytoplasm during root nodule development [29,30].

Emerging evidence showed that many lncRNAs also participate in response to several stress conditions in plants [18,31,32,33]. Indeed, field crops are continuously exposed to a combination of different biotic and abiotic stresses. To reduce these damages, plants have evolved sophisticated adaptive response mechanisms to reprogram its gene expression at the transcriptional, post-transcriptional and post-translational levels [15]. At the molecular level, it has been reported that the response of plants to a combination of two different abiotic stresses cannot be directly extrapolated from the response of the plant to each of the different stresses applied individually. Plant responses to multiple stress exposures were studied, either in combination or individually using large-scale microarrays analysis. This revealed that 60% of the protein coding transcriptome changes in response to double stress was not predictable from the responses to single stress treatment [34], suggesting different transcriptional responses to different combined stress exposure.

Maize is one of the most important food crops in the world, with a harvested area estimated in about 140 million ha (FAO 2012). Biotic and abiotic stresses are important limiting factor for yield and grain quality in maize production. Lluteño maize (*Z. mays* L. amylacea) is a Chilean local race of sweet corn developed by the first native farmers of the Lluta Valley, Atacama Desert (Arica, Chile) [35], and still maintained by traditional farmers even with the introduction of commercial hybrid lines. The soils of the Lluta Valley have high levels of salinity (EC_iw_ = 2.2–5.9 dSm^−1^) and boron concentration (10–29 ppm) [36]. Elevated levels of salinity and boron naturally occurring in soil and irrigation water are detrimental to several crops grown in agricultural regions, especially those frequently associated with arid and semi-arid environments [37,38,39]. Lluteño maize has evolved to successfully thrive in hyper-arid habitats, suggesting an inherent tolerance to salinity, and high levels of boron, among other elements [35]. This ability makes the Lluteño maize a good system to identify the genetic basis and understanding adaptive response mechanisms, which govern the salt and boron stress tolerance in a local maize landrace from northern Chile.

Microarrays and RNA sequencing approaches have been successfully employed to determine the transcriptional response to either salinity or boron in plants [40,41,42]. However, the combined effect of both stresses over the plant transcriptional activity has not been analyzed. In this study, we performed transcriptional profiling to identify responsive lncRNAs in Lluteño maize under stress induced by the combined effect of high salinity and boron. We identified a total of 1710 salt and boron stress responsive lncRNAs; of which 755 seem to be absent in three maize commercial variants with genome sequence available. In general, Lluteño exclusive lncRNAs are preferable expressed in roots under a long-time exposure to stressed conditions and presented an unusual higher expression levels in roots under long-time exposure to stressed conditions compared to protein coding genes. We also identified a set of 848 stress responsive potential *trans* natural antisense transcripts (*trans*-NAT) lncRNAs, which seems to be regulating genes associated with regulation of transcription, response to stress, response to abiotic stimulus and participating of the nicotianamine metabolic process. We experimentally validated 12 responsive maize lncRNAs using reverse transcription-quantitative PCR (RT-qPCR). Their altered expression levels were confirmed in presence of salinity and high boron concentrations, suggesting that at least a subset of these newly identified Lluteño lncRNAs may play important roles in response to salinity and boron stresses in maize.

## 2. Materials and Methods

### 2.1. Plant Growth and Stress Treatment

Lluteño maize (*Z. mays* L. cv. amylacea) and two commercial hybrid (Prays-214 and GH-2041) seeds were germinated in perlite under greenhouse conditions for two weeks. These seedlings were transferred to hydroponic culture with Hoagland’s solution (KNO_3_ (6 mmol L^−1^), Ca(NO_3_)_2_ (4 mmol L^−1^), NH_4_H_2_PO_4_ (1 mmol L^−1^), MgSO_4_ (1 mmol L^−1^), H_3_BO_3_ (25 µmol L^−1^), MnSO_4_ (2 µmol L^−1^), ZnSO_4_ (2 µmol L^−1^), CuSO_4_ (0.5 µmol L^−1^), (NH_4_)_2_MoO_4_ (0.5 µmol L^−1^) and Fe-EDDHA (20 µmol L^−1^)), which was renewed every 3 days. After 10 days of acclimatization, seedlings were exposed to 150 mM NaCl and 20 ppm of B for 3 and 96 h. The control samples (non-stressed plants) were collected at the same time of the samples with 3 h of stress. Roots and leaves samples from three plants were collected in pool, frozen in liquid nitrogen and immediately stored at −80 °C until RNA isolation.

### 2.2. Ion Analysis

To determine the mineral elements (Na^+^ and B) contents, leaves and roots samples were dried at 60 °C and finely ground. Then, were dry-ashed in a muffle furnace at 550 °C for 4 h and treated with HCl (2 N). The Na^+^ content was quantified using a flame photometer (Model PFP7, Jenway, Staffordshire, UK). The B content was quantified using a molecular absorption spectrometer (Model UV-2100, Unico, NJ, USA). Ion content was measured in pools of leaves and roots of two plants, in three biological replicates, with three additional technical replicates for each biological replicate.

### 2.3. RNA Extraction and Sequencing

Total RNA from leaves and roots tissues were isolated using the Trizol reagent (Invitrogen, Carlsbad, CA, USA), according to procedures specified by the manufacturer. The extracted RNA was re-suspended in 20 µL of RNase-free water and stored at −80 °C. Integrity was verified by electrophoresis in 1% agarose gels. RNA was purified with the RNeasy MinElute Cleanup Kit (Qiagen, Hilden, Germany), according to procedures specified by the manufacturer. Then, 1 µg of RNA were treated with DNase I in a final volume of 10 µL and incubated at 37 °C for 1 h. The reaction was stopped by adding 1 µL of ethylenediaminetetraacetic acid (EDTA) (25 mM) at 65 °C for 5 min. The total RNA was used to prepare oligo-dT Illumina paired-end shotgun libraries using the Nextera kit (Illumina, San Diego, CA, USA). The libraries were sequenced using the Illumina MiSeq platform (150 bp paired-end reads). All raw data generated were deposited in Sequence Read Archive (SRA) National Center for Biotechnology Information (NCBI) database with the accession number SRP077718.

### 2.4. Raw Data Filtering and Quality Control

The quality control of sequenced reads consisted in the trimming, followed by the filtering of low quality, low complexity reads and the elimination of sequencing contaminant sequences. Using PRINSEQ (version 0.20.4) [43], we trimmed the extremity of raw reads (5 bp for 5’ and 15 bp for 3’) and removed those with a Phred quality score mean below 28. Low complexity reads were eliminated using the options “−lc_method entropy, −lc_threshold 0, 16 and 26” for homopolymers, dinucleotides and trinucleotides repeats, respectively. We used the software Bowtie2 (version 2.3.0) [44] to discard reads that aligned against chloroplast, ribosomal and mitochondrial sequences available in the GenBank database [45]. The resulting sequences were considered as filtered and high-quality reads, which were used in further analysis.

### 2.5. Transcriptome Reconstruction Strategy and Long Non-Coding RNAs Identification

To identify all long non-coding RNAs expressed in Lluteño maize, we implemented a hybrid strategy through a mix of de novo and genome reference-based transcriptome assembly. Firstly, in order to obtain all transcriptional fragments, we used the filtered and high quality reads in two strategies, using (a) the Trinity de novo assembly protocol [46] and (b) the TopHat/Cufflinks genome reference based protocol [47], using B73 maize genome sequence as reference [48]. Trinity isoforms were clustered using CD-HIT-EST [49] with 90% identity to eliminate redundancies and generate de novo transcriptional fragments; while genome-based transcripts were clustered based on genomic coordinates using cuffmerge, from Cufflinks package. Finally, both de novo and genome-based transcripts were clustered together using CD-HIT-EST, with 90% of global sequence identity. The quality of this assembling strategy was evaluated according to the length of final transcripts and the re-mapping of original reads using Bowtie2 (version 2.2.4) [44]. At this point, an additional contaminant filtering was applied using Nucleotide Basic Local Alignment Search Tool (BLASTn) [50] searches against NCBI nucleotides (NT) database [45]. The results were visualized using the software MEGAN5 (version 5.6.3) [51]. Transcripts that aligned with plantae distantly related organisms (i.e., fungi, proteobacteria, protozoa, and amoebozoa) were discarded. The resulting dataset was considered as the first reference transcriptome of Lluteño maize.

To identify the non-coding transcripts, we firstly aligned the reference transcriptome using BLASTx against the NCBI/NR protein database and the UniProt/SwissProt protein database [52]. Sequences matching with proteins were excluded from further analysis. The remaining sequences had their coding potential evaluated using Transdecoder (version 2.0) [46], CPC (version 1) [53] and ESTScan (version 2.1) [54]. If a transcript was predicted as potential coding by at least one of the tools, it was considered as a potential protein coding or pseudogene and excluded from the final dataset. Resulting sequences smaller than 200 nt were considered Lluteño maize small non-coding RNAs, while those bigger or equal than 200 nt were considered long non-coding RNAs. To identify the lncRNAs as potential miRNA precursors, we performed sequence-similarity searches using Bowtie1 (version 1.1.1) [55], allowing a unique mismatch. All miRNA sequences available on the Non-coding RNA Databases Resource [4] were used and mapped against all Lluteño dataset of lncRNAs.

### 2.6. Comparative Genomics of Lluteño Long Non-Coding RNAs

To identify the relationship of Lluteño maize with other commercial variants, we performed different sequence similarity searches against the genomic sequences from maize with genome data available at MaizeGDB [48]. For B73, the most used and completely sequenced maize variant, we used the genome assembly release RefGen_v3. For Palomero, we used the assembly version v1, containing 196,697 contigs. For Mo17, the genome sequence is not assembled, and we used all reads available (total of ~63 million). The mappings against assembled genomes (B73 and Palomero) were performed using BLAT (version 3.2.1) [56], while the mapping between the reads from Mo17 against Lluteño lncRNAs was performed using Bowtie2 [44]. Non-coding RNAs that did not present any match with B73, Palomero or Mo17 sequences were considered as Lluteño exclusive.

Additionally, to identify the conservation of Lluteño lncRNAs with known transcripts from maize publicly available lncRNAs, we used BLAT (75% mutual coverage, 90% identity) to map our dataset against the list of long non-coding transcripts described by Wang et al. [57], Boerner and McGinnis [58], Zhang et al. [33] and Li et al. [17].

### 2.7. Differential Expression Analysis

To identify statistically differentially expressed long non-coding RNAs among different tissues and treatments, genomic coordinates files in SAM format were generated by the mapping of high quality reads against the assembled non-coding transcripts using Bowtie2. Raw counts per transcripts were quantified using HTSeq-count (version 0.5.4) [59]. Finally, the edgeR package (version 3.8.0) [60], an R-based tool within the Bioconductor project, was used for the detection of changes in abundance based on those counts. Counts were normalized by Trimmed Mean of M-values (TMM) method and filtered by a four-fold change cutoff (up- or down-regulated, *p*-value < 0.001) in each comparison to be considered as differentially expressed transcript. To generate a visual figure of differentially expressed lncRNAs, we generated a hierarchical clustering on library mean normalized read counts and plotted in a heat map representation using the R package gplots (version 3.0.1) [61].

### 2.8. Quantitative Reverse Transcription Polymerase Chain Reaction Validation

The synthesis of the first cDNA strand was performed from 1 µg total RNA. The RNA treated with DNase I (Invitrogen), together with 3 µL oligo dT (10 pmol/µL) and 3 µL dNTP’s (2.5 mM) in a volume of 17 µL, was incubated at 65 °C for 5 min. Then, 8 µL of 5× First Strand Buffer (Invitrogen), 2 µL 0.1 M dithiothreitol (DTT), 1 µL RNasaOut (Invitrogen) and 1 mL SuperScript III reverse transcriptase (Invitrogen) were added in a final volume of 40 µL. Reverse transcription was performed in an Amplitronyx A6 (ATC401) Thermal Cycler (Nyx Technik, Inc., San Diego, CA, USA), using the following program: 15 °C for 10 min, 25 °C for 16 min, 42 °C for 60 min, 70 °C for 10 min and 4 °C final hold. The tubes were stored at −20 °C until further use.

For quantification analysis, the transcripts of interest were amplified using the Maxima SYBR Green/ROX qPCR Master Mix (2×) (Thermo Scientific, Waltham, MA, USA) and real-time PCR analyses were performed on an Eco Real-Time System (Illumina). Maize *CUL* gene was used as housekeeping gene for normalization [62]. The reaction conditions used were as follows: 95 °C for 10 min, 40 cycles of 95 °C for 15 s, 60 °C for 15 s, 72 °C for 15 s, and an analysis of dissociation or melting of 55 to 95 °C with a temperature increase of 0.3 °C s^−1^.

The efficiency of the PCR reaction in real time was calculated for each one of the transcripts by using 6 serial dilutions of complementary DNA (cDNA), with an initial concentration of 32 ng and a dilution factor of 1:2. The efficiency was determined with the following formula: Efficiency (E) = 10 ^(−1/slope)^ − 1. The primers used in our PCR experiments are described in Table S1.

### 2.9. Quantification and Data Analysis of Reverse Transcription-Quantitative PCR Products

Considering an efficiency of about 100% in all reactions, the quantification method used to measure the relative changes in genes expression was 2^−ΔΔCt^, based on Livak and Schmittgen [63]. Three biological replicates and three technical replicates for each biological replicate were used.

### 2.10. Trans Natural Antisense Transcripts Analysis

The identification of potential *trans*-NATs was performed following the same strategy developed by Wang and collaborators [64]. All lncRNA transcripts were aligned against themselves and against all coding genes available on the reference B73 genome using BLASTn [65]. Similar to Wang and colleagues, if paired regions within transcripts covered more than half of the length within them, both were considered as a *trans*-NAT pair and classified as ‘high-coverage’ *trans*-NAT. Otherwise, if the matching between two transcripts has a continuous pairing region longer than 100 nucleotides, they were classified as ‘100 nt’ pair. Gene ontology (GO) enrichment analysis (*p* < 0.05) of target genes was performed through AgriGO [66], using the coding genes from B73 as reference.

## 3. Results

### 3.1. Lluteño Maize Is Extremely Resistant to Salt and Boron Compared to Commercial Hybrids

**Figure 1:**
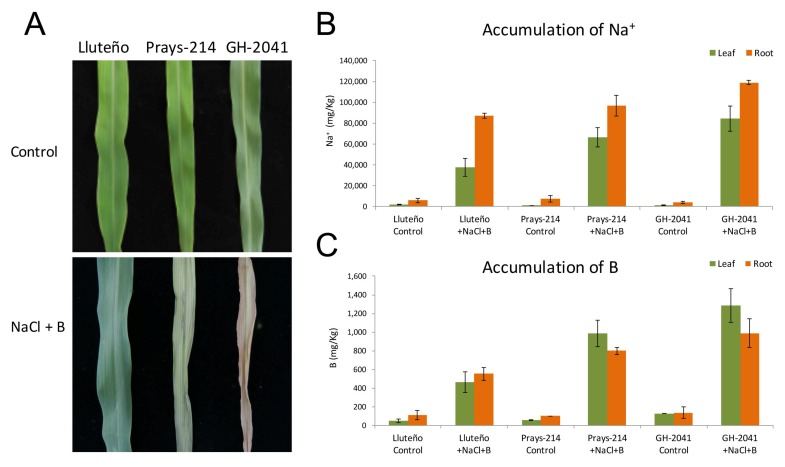
Responses of Lluteño maize to salinity and boron stress. (**A**) differential responses of Lluteño and the commercial hybrids Prays-214 and GH-2041 seedling to salinity and boron stress after 10 days; (**B**) Na^+^ accumulation of Lluteño maize seedling under 150 mM NaCl and 20 ppm B; and (**C**) B accumulation of Lluteño maize seedling under 150 mM NaCl and 20 ppm B.

Two commercial maize hybrids widely cultivated in the north of Chile (cultivar Prays-214 and cultivar GH-2041), and the landrace Lluteño maize, were grown in hydroponic solution for 10 days, and then exposed to 150 mM NaCl and 20 ppm B. Under control conditions it was not possible to observe any stress symptoms in the three maize varieties (Figure 1A). However, during the first days of exposure to NaCl and B, Prays-214 and GH-2041 cultivars started the development of stressed symptoms in leaves, which gradually became yellow, with necrotic lesions at the leaf tip. After 10 days, both commercial cultivars were drastically affected (Figure 1A). In contrast, during the first days after stress exposure, Lluteño maize showed a decreasing in chlorophyll content but morphological effects induced by these stresses were not observed (Figure 1A). To determine the distribution of Na^+^ and B under our experimental conditions, the content of these ions in roots and leaves were measured at 96 h after stress exposure. In general, a compartmenting of Na^+^ was observed, with a preference for accumulating Na^+^ in roots compared to leaves. The preferential accumulation is most accentuated in Lluteño maize than commercial cultivars (Figure 1B). The Na^+^ content in roots was slowly minor in Lluteño maize than commercial hybrids. However, the Na^+^ content in leaves is much lower in Lluteño maize compared to Prays-214 and GH-2041 (Figure 1B). Lluteño landrace presented a Na^+^ content relation between roots and leaves (root/leaves) of 2.25, while this relation was of 1.33 and 1.25 for Prays-214 and GH-2041, respectively. Regarding the content of B, differences in B accumulation between roots and leaves in all three maize varieties were not observed. However, Lluteño maize accumulates approximately half the B content in their tissues as the commercial hybrids (Figure 1C).

### 3.2. Identifying a Comprehensive Repertoire of lncRNAs in Lluteño Maize Transcriptome

**Figure 2:**
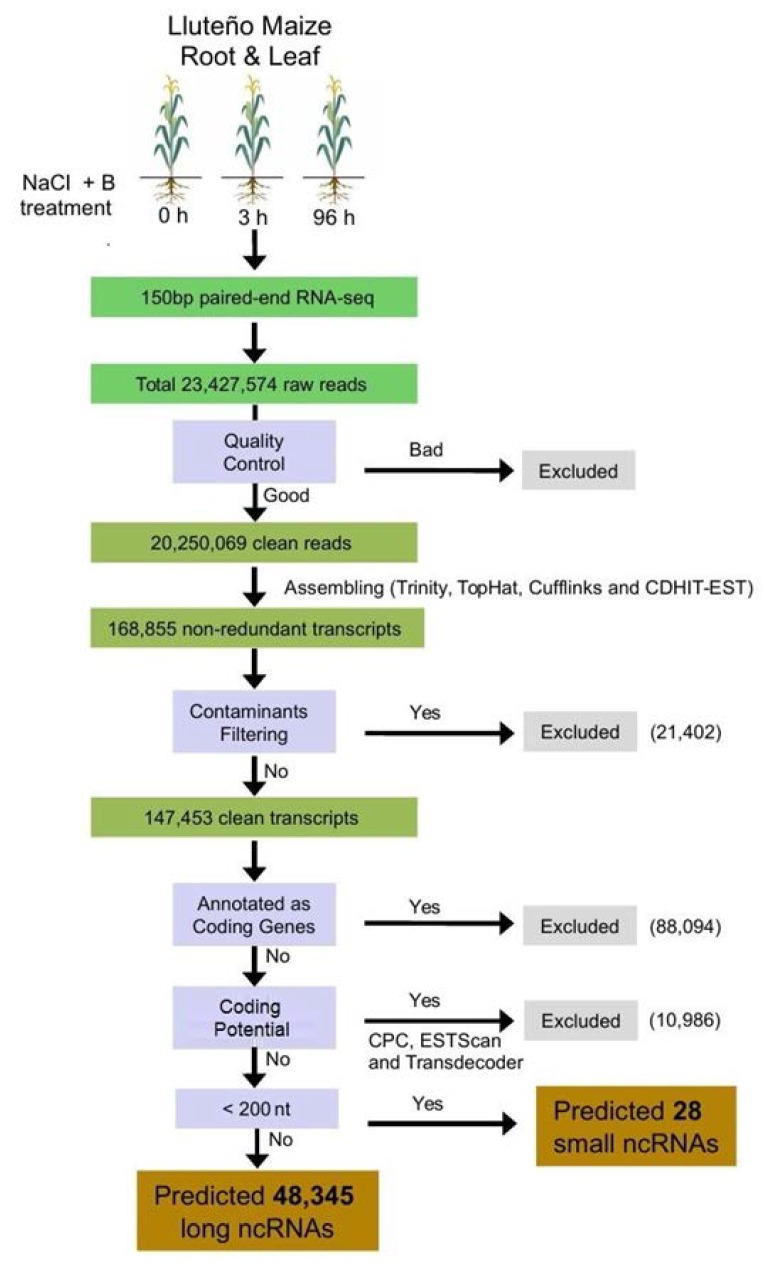
General flowchart of the pipeline used to identify the repertoire of long non-coding RNAs (lncRNAs) from Lluteño maize expressed under to salt and boron stress conditions. RNA-seq: RNA sequencing.

High quality reads obtained from our sequencing runs were used as input to our hybrid transcriptome assembling strategy, resulting in a total of 168,855 non-redundant transcripts expressed on Lluteño maize. Considering this high number of transcripts expressed under salt and boron conditions in Luteño maize, we performed an extra procedure of contaminants filtering using BLASTn searches against NCBI NT database. This analysis identified 21,402 transcripts from several classical soil biological contaminants such as fungi, amoeba and bacteria. The general work chart in Figure 2 details the analysis performed to identify the non-coding RNAs repertoire present on the dataset of all expressed transcripts. There are many transcripts that have similarities with coding genes that requires further characterization, but it is not the focus of this report. Moreover, we identified nearby ten thousand mRNAs or pseudogenes with a potential to code for peptides, which were predicted by sequence similarity searches using BLASTx against protein sequences, or had their coding potential calculated using ESTScan2, CPC or Transdecoder. Finally, excluding all transcripts with the potential to be translated into peptides, we identified a total of 48,345 long non-coding RNAs, presenting an average length of 309 nucleotides. This number of transcripts is much bigger than the previously identified for maize hybrids [17,33,57,58], suggesting a potential spatial–temporal divergence between Lluteño and other maize hybrids. Due to the high number of transcripts generated using a relatively small number of reads (22.2 millions), compared to an Illumina High-Seq run, we observed that using the half part of generated reads we were able to reconstruct about 90% of ncRNA transcripts (Figure S1). This analysis suggests that the inclusion of novel reads would not increase significantly the number of identified lncRNAs, but it could be useful for the identification of lncRNAs with lower expression levels.

It is known that secondary structure is an additional feature for the functional characterization of non-coding RNAs [67]. Using the AlifoldZ approach [68], we found that 31,109 lncRNAs (64.3% of total) possess RNA structures presenting significant low energy compared to sets of random sequences (negative z-score, Figure S2), forming stable structural domains that could be important for their processing or biological function. However, the absence of stable secondary structures is not necessarily an indicative of lack of function. There are several known plants ncRNAs that can act as natural antisense transcripts, achieving their functional roles by base pairing a target RNA [12].

Moreover, it is well documented in the literature that small regulatory RNAs can be generated by the processing of long RNAs precursors [69]. To ask what fraction of our set of lncRNAs expressed in Lluteño maize could be precursor of small RNAs, we compared their sequences to those of known miRNAs available in public databases [4]. Only a discrete overlap was found, with 170 potential miRNA precursors identified, indicating that these long transcripts are predominantly not precursors of known miRNAs, yet leaving open the possibility that these transcripts could represent precursors of uncharacterized novel small RNAs.

### 3.3. Lluteño Maize Presents a Surprisingly Number of Exclusive lncRNAs

**Figure 3:**
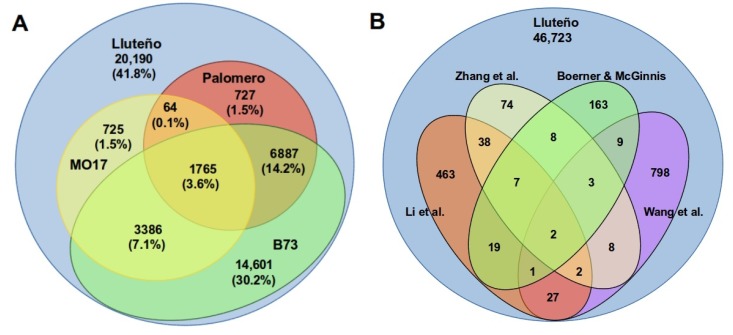
(**A**) Comparative RNomics of Lluteño lncRNAs in relation to four different maize hybrids with genome sequence available: MO17, Palomero and B73; (**B**) Lluteño lncRNAs also available in other four different works that previously characterized different datasets of long non-coding RNAs in *Zea mays*. References: Zhang et al. [33], Boerner and McGinnis [58], Li et al. [17] and Wang et al. [57].

To determinate the conservation of the repertoire of Luteño lncRNAs expressed under exposure to salt and boron with other maize hybrids, we aligned all lncRNAs transcripts against other maize genome sequences available in literature (Figure 3A). This analysis revealed that 28,012 (58.1%) lncRNAs are conserved at a genomic level (90% coverage) with other maize (B73, Mo17 or Palomero), with the remaining 41.8% belonging to potentially Lluteño exclusive lncRNA transcripts. From the total of Lluteño lncRNAs conserved with other *Z. mays* hybrids, 12,047 (25% out of the total lncRNAs) were identified as conserved with at least two different hybrids, while only 1749 lncRNAs determined to be shared by all the four hybrids under study: Lluteño, B73, Mo17 and Palomero. It is important to note that the higher conservation was observed with B73 (14,529, 30.15% of the total). This might be associated with the fact that B73 is the reference genome for *Z. mays* and, consequently, presents the more complete assembly in comparison to other maize with genome sequences available. However, 1509 (3.13%, of the total) conserved lncRNAs were not detected in B73, being shared with Mo17 or Palomero. 

In addition to the conservation analysis with other maize hybrids at a genomic level, we also downloaded other lncRNAs reported to *Z. mays* in the scientific literature to identify the set of conserved transcripts (Figure 3B). This search showed 1622 (3.4% of the total) conserved lncRNAs between our set of Lluteño long non-coding RNAs and public available non-coding transcripts (75% of coverage between transcripts). Approximately 52.4% (850 out of 1622) of conserved transcripts are present in the study of Wang and collaborators [57], while 34.5% (559 out of 1622) of conserved transcripts are present in the study of Li and collaborators [17], and 13% (212 out of 1622) were present in the study of Boerner and McGinnis [58]. In comparison with the work of Zhang and collaborators [33], we identified only 142 conserved transcripts.

### 3.4. Genomic Organization of Lluteño lncRNAs According to B73 Reference Sequence

**Figure 4:**
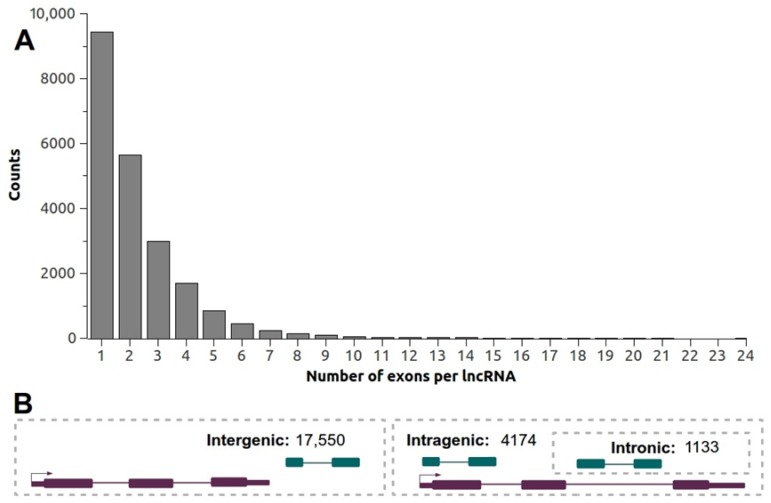
(**A**) Distribution of the number of exons per lncRNA obtained by the mapping Lluteño lncRNAs against B73 variant; (**B**) General overview of the genomic organization and structure of Lluteño lncRNAs according to B73 genome sequence. Purple blocks and lines represent protein coding genes, while dark green blocks and lines represents lncRNAs.

To estimate the genomic organization and distribution of Lluteño lncRNAs, we used the hybrid B73 genome sequence as a reference. In total, 55.1% (26,639) of Lluteño lncRNAs mapped to B73 genome. Of these, 21,724 (81.6%) mapped only one time on the genome, while the remaining 4915 (18.4%) mapped multiple times. According to the non-coding transcripts that mapped uniquely on the genome, 9425 (43.3%) were classified as mono-exonic or unspliced lncRNAs, while 12,299 (56.7%) were classified as multi-exonic or spliced lncRNAs. The lncRNAs presented an average number of 3.2 exons per transcripts (excluding unspliced transcripts), with the average length of these exons estimated in 144.08 nt, and the average length of its intronic regions estimated in 661.58 nt. Figure 4A shows the distribution of the exons number per lncRNAs. In total, 116 lncRNAs are estimated to possess more than 10 exons, with one of them containing 24.

According to its genomic localization (Figure 4B) relative to B73 known protein coding genes, we identified a considerable number of lncRNAs (4174, 19.21% of uniquely mapped lncRNAs) potentially originated from intragenic regions of the genome, with 1133 (5.21%) originated from a totally intronic regions (Figure 4B). It is important to note that we do not have the orientation of these transcripts to characterize them as sense or antisense lncRNAs. On the other hand, the majorities of the non-coding transcripts (17,550, 80.79% of uniquely mapped lncRNAs) are potentially originated from intergenic regions and could be classified as lincRNAs (Figure 4B). Regarding the chromosomal distribution of Lluteño lncRNAs in B73 genome, we observed a very similar frequency distribution of our lncRNAs compared to B73 protein coding genes along all chromosomes (Table S2). Finally, we investigated if these lncRNAs could be regulating their neighbor coding genes. For that, we calculated the Pearson correlation between the expressions of all lncRNAs located within 10 kilobases up/downstream of protein coding genes (Table S3). We found 4660 lncRNAs containing at least one coding gene located up to 10 kb. Of these, 1672 presented a negative correlation (502 ≤ −0.5); 236 do not have correlation; and 2752 presented a positive correlation (1775 ≥ 0.5).

### 3.5. An Overall Higher Expression of Long Non-Coding RNAs Compared to Protein Coding Genes Is Observed in Lluteño Maize under 96 h of Exposure to Stressed Conditions

**Figure 5:**
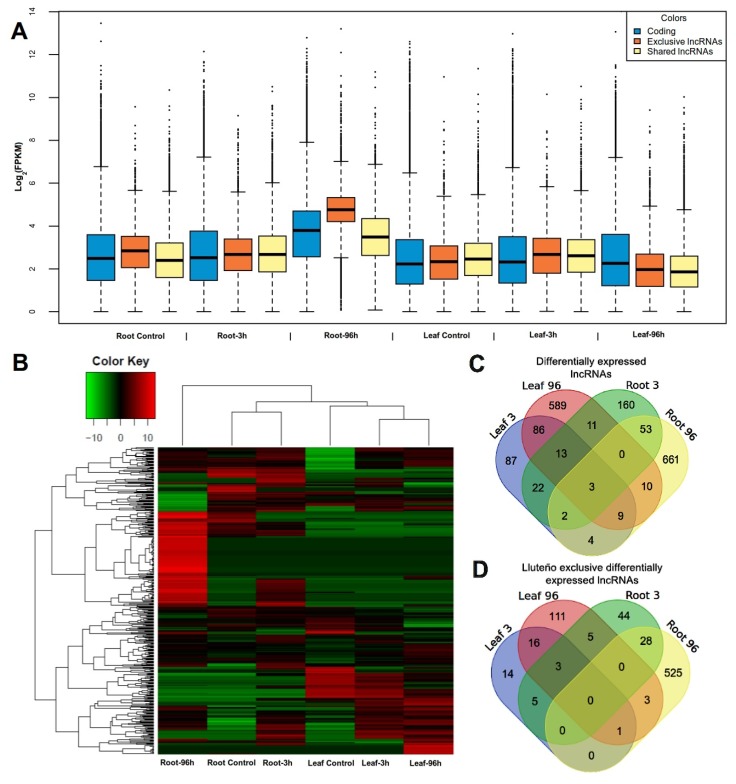
Differentially expressed lncRNAs in Lluteño maize. (**A**) Comparison of expression values log_2_ fragments per kilobase million (log_2_FPKM) between each condition. Blue boxes are coding genes, orange boxes are Lluteño exclusive lncRNAs and light yellow are the lncRNAs shared with other *Z. mays* hybrids. The boxes represent 50% of the expressed transcripts, while dots above and below the boxes represents the other 25% of the transcripts with higher and smaller expression levels, respectively. The boxes size represents the expression levels variability of the transcripts. A cutoff of 1 FPKM was used to define if a transcript is expressed or not; (**B**) Heat map representation in logarithmic scale of library mean normalized read counts of differentially expressed lncRNAs (fold change higher than four); (**C**,**D**) Venn diagrams of the comparison between differentially expressed lncRNAs in each time condition, all lncRNAs and Lluteño exclusive lncRNAs respectively. All conditions were compared against its same tissue at 0 h (control) of salt and boron exposure.

It is known that long non-coding RNAs are in general expressed at lower levels compared to protein coding genes [70,71]. To evaluate the expression levels of Lluteño maize lncRNAs and coding genes, we compared the fragments per kilobase million (FPKM) overall expression levels of both type of transcripts in all conditions (control, 3 h, 96 h of exposure to salt and boron) from roots and leaves samples (Figure 5A). When comparing the expression levels of both sets of exclusive (orange boxes) and shared (yellow boxes) lncRNAs with protein coding genes (blue boxes), it is possible to observe an overall slightly lower expression of lncRNAs compared to coding genes (Figure 5A). However, this analysis showed an unusual higher expression levels of Lluteño exclusive lncRNAs in roots under long exposure to stressed conditions (96 h) compared to protein coding genes and the set of lncRNAs shared with other maize hybrids. These results may suggest a potential adaptability of Lluteño maize to surpass the stressed environment of Atacama Desert. The boxes size in Figure 5A represents the expression variability of the transcripts. As one can observe, Lluteño lncRNAs (exclusive and shared) presented a similar variability in their expression levels in almost all conditions, with exception to that set of exclusive lncRNAs under 96 h of exposure to stressed conditions, which presented a lower variability (smaller boxes).

### 3.6. Long Non-Coding RNAs Are Differentially Expressed Especially on Roots under Long Exposure to Salt and Boron

To gain further insights on the putative biological relevance of long non-coding RNAs in response to salt and boron stress, we investigated their relative expression on roots and leaves tissues under control, 3 and 96 h of exposure. Of the total 48,345 non-coding transcripts, 10,950 (22.6%) presented as expressed in all treated conditions. A total of 5889 (12.2%) were detected as expressed exclusively on control, while 3495 (7.2%) and 14,483 (30%) on three and ninety-six h of exposure to stressed conditions (Figure S3). Differential expression analysis revealed a total of 1710 salt and boron stress responsive lncRNAs (Figure 5B,C), of which 755 seem to be absent in the other three maize variants (Figure 5D). According to the differentially expressed lncRNAs shared with the reference B73 genome (748 in total), 130 mapped within intragenic regions, with 102 of them presenting overlap with coding genes (exonic, 5’ untranslated region, (5’UTR) or 3’UTR). The absence of oriented libraries precludes us from being sure whether these 102 lncRNAs are sense-overlapping non-coding RNAs or if they are transcribed from the antisense strand. All expression values, fold-changes for each condition and its presence in other maize are available in the Supplementary Materials, Table S4. Interestingly, approximately 64.7% of statistically differentially expressed lncRNAs in roots are Lluteño exclusive (Figure 5B–D), while, in leaves, about 81.1% of differentially expressed lncRNAs are shared with other *Z. mays* hybrids. Few lncRNAs (74) showed to be statistically differentially expressed on roots and leaves simultaneously.

In this analysis, we were also able to identify three different sets of lncRNAs associated with an early, late and prolonged response to stressed conditions (Figure 5C,D). We defined as an early response those lncRNAs differentially expressed only under 3 h of exposure compared to control; late response those detected as differentially expressed after 3 and up to 96 h of exposure compared to control; and as prolonged response those lncRNAs differentially expressed at both 3 and 96 h of exposure compared to control. In an early response, we identified 160 lncRNAs associated with this condition in roots, of which 27% were exclusive to Lluteño maize. In leaves, we identified 87 lncRNAs associated with an early response to stressed conditions, with 16% exclusive to Lluteño maize. In a late response, we identified 661 non-coding transcripts in roots, with a high incidence of Lluteño exclusive lncRNAs, counting 79% of all the late responsive lncRNAs. The opposite was observed in leaves, in which we identified 589 late responsive non-coding transcripts, with only 18% exclusive to Lluteño maize. Finally, with a prolonged response to stressed conditions we identified 53 lncRNAs in roots (52% Lluteño exclusive); and 86 lncRNAs in leaves (18% Lluteño exclusive). Therefore, these results may suggest a potential mechanism of defense to surpass the stressed condition. 

### 3.7. Validation of Novel lncRNAs by Quantitative PCR

**Figure 6:**
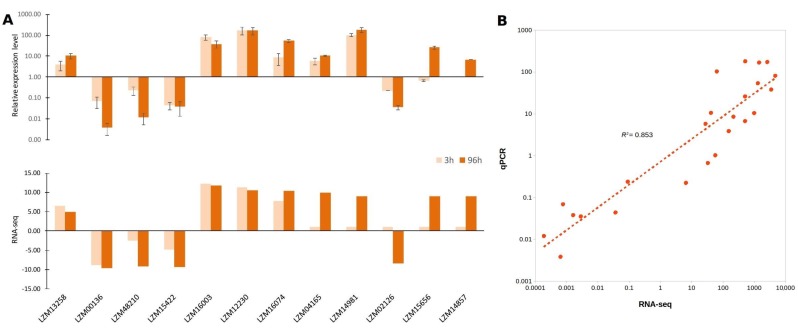
Validation of RNA sequening (RNA-seq) results by reverse transcription-quantitative PCR (RT-qPCR) of the differentially expressed lncRNAs in response to salt and boron stress. (**A**) Comparisons between qPCR validation, compared to RNA-seq. The upper graphic represents the differential expression analysis of 10 leaves and 2 roots randomly selected lncRNAs at 3 and 96 h after exposure to 150 mM NaCl and 20 ppm B stress conditions obtained by RT-qPCR. The lower graphic represents the fold changes based on FPKM calculated from globally normalized RNA-seq data. For RT-qPCR, the Cullin gene was used on the normalization of gene expression. The values are presented as an average and standard error (SE) of three independent experiments, with three technical replicates; (**B**) Correlation analysis according to qPCR and RNA-seq assays.

To further validate the expression of these salt and boron responsive lncRNAs, RT-qPCR was performed to detect the expression profile of 12 randomly selected lncRNAs out of 1710, at 3 and 96 h after stress exposure (Figure 6A). We evaluated the activity of 10 and 2 lncRNAs differentially expressed in leave and roots respectively. Similar to RNA sequencing (RNA-seq) data, the RT-qPCR results showed that almost all lncRNAs were successfully validated in stressed roots and leaves at 96 h. The only exception was the leaves lncRNA LZM02126, at 3 h after stress exposure. Both RT-qPCR and RNA-seq assays presented an expression fold-change positive correlation with an *R^2^* of 0.853 (Figure 6B). These findings confirm that these lncRNAs are responsive to the combined salt and boron stress in maize leaves and roots under different hours of exposure.

### 3.8. Long Non-Coding RNAs as Potential Trans Natural Antisense Transcripts 

**Figure 7:**
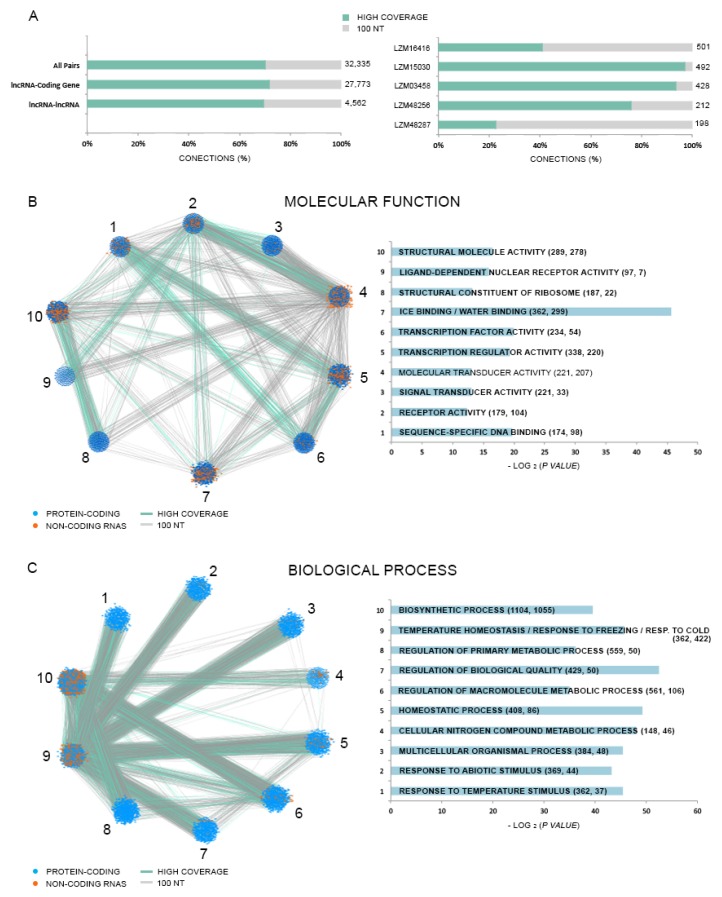
(**A**) On the left, we have the distribution of *trans* natural transcript (*trans*-NAT) pairs according to its classification (high-coverage or 100 nt), and the target transcripts type (coding genes or lncRNAs). On the right, we have the top five lncRNAs with the most target transcripts; (**B**,**C**) Network and histogram of the top ten ‘molecular function’ and ‘biological process’ enriched Gene Ontology (GO) categories, respectively.

We predicted a total of 32,335 *trans*-NAT pairs occurring within Lluteño lncRNAs (Figure 7A), including 27,773 lncRNA-coding gene pairs and 4562 lncRNA-lncRNA pairs. Of these, 22,736 (19,423 lncRNA-coding gene; 3313 lncRNA–lncRNA) were classified as ’high-coverage‘ pairs according to the definition of Wang and collaborators [64], and 9599 (8350 lncRNA-coding gene; 1249 lncRNA-lncRNA) as ‘‘100 nt pairs’’. The lncRNA with the biggest number of pairs is LZM16416, with 501 pairs. The top five lncRNAs with the most pairs are listed in Figure 7A. Two *trans*-NAT networks were constructed based on the observed pairs and gene ontology enrichment analyses, using the top ten enriched categories according to ‘molecular function’ and ‘biological process’ GO categories (Figure 7B,C, respectively). We identified 11 enriched (*p* < 0.05) categories related to ‘molecular function’ and 46 related to ‘biological process’ GO categories (Table S5). The top five ‘molecular function’ enriched terms were: ‘sequence-specific DNA binding’ (98 lncRNAs associated with 174 coding genes), ‘receptor activity’ (104 lncRNAs associated with 179 coding genes); ‘signal transducer activity’ (33 lncRNAs associated with 221 coding genes); ‘molecular transducer activity’ (207 lncRNAs associated with 221 coding genes); and ‘transcription regulator activity’ (220 lncRNAs associated with 338 coding genes). The enriched categories containing the higher number of lncRNAs are: ‘ice binding’ and ‘water binding’, with 299 lncRNAs pairing 362 coding genes; ‘structural molecule activity’, with 278 lncRNAs pairing 289 coding genes; and ‘transcription regulator activity’, with 220 lncRNAs pairing 338 coding genes. On the other hand, the top five ‘biological process’ enriched terms were: ‘response to temperature stimulus’ (37 lncRNAs associated with 362 coding genes), ‘response to abiotic stimulus’ (44 lncRNAs associated with 369 coding genes); ‘multicellular organismal process’ (48 lncRNAs associated with 384 coding genes); ‘cellular nitrogen compound metabolic process’ (46 lncRNAs associated with 148 coding genes); and ‘homeostatic process’ (86 lncRNAs associated with 408 coding genes). The enriched categories containing the higher number of lncRNAs are: ‘biosynthetic process’, 1055 lncRNAs pairing 1104 coding genes; ‘temperature homeostasis’, ‘response to freezing’ and ‘response to cold’, with 422 lncRNAs pairing 362 coding genes; and ‘regulation of macromolecular process’, with 106 lncRNAs pairing with 561 coding genes. These results suggest that at least a set of Lluteño *trans*-NAT lncRNAs could be regulating genes associated to the response to stressed conditions.

**Figure 8:**
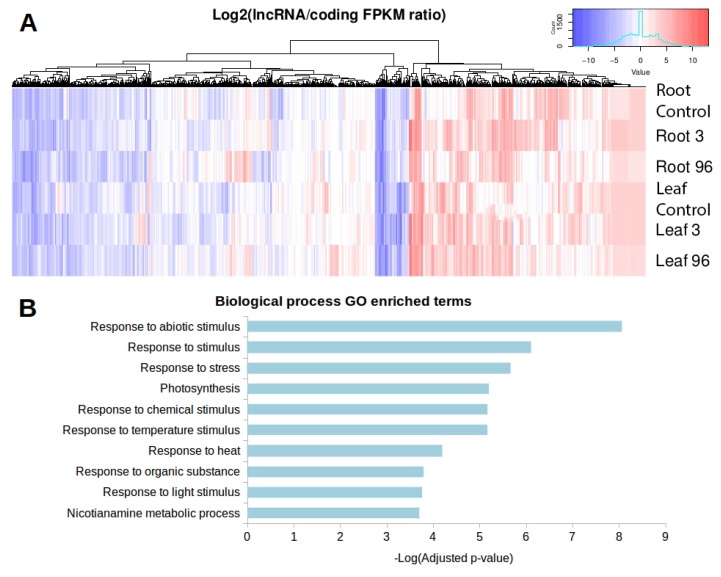
(**A**) Heat map representation of the expression ratio between differentially expressed *trans*-NAT lncRNAs and their respective target coding gene. In red, we have the pairs in which the lncRNA is overexpressed compared to their paired coding gene. In blue, the coding genes are overexpressed compared to their paired lncRNA; (**B**) ‘Biological process’ Gene Ontology enriched categories of differentially expressed *trans*-NAT lncRNAs.

The functionality of *trans*-NATsis directly associated with the co-expression of both transcripts, expressed at the same cell or condition, allowing the formation of double-stranded RNA duplexes. We evaluated the expression of lncRNA-coding gene pairs in all studied conditions from our RNA-seq approach (Table 1). The condition with the most transcripts in the co-expressed pair is leaves under 96 h of exposure, with 10,842 pairs. On the other hand, leaves at 96 h is the condition with the fewest co-expressed pairs. We identified that 848 *trans*-NAT lncRNAs, containing 1959 lncRNA-coding gene pairs, presented as differentially expressed in response to salt and boron exposure. We plotted the lncRNA/coding gene expression ratio to identify the distribution of which transcripts, lncRNAs or coding genes, are normally overexpressed in the pairs (Figure 8A). The heat map shows a clear separation of three groups of *trans*-NATs according to the lncRNA/coding gene ratio. The left-side group is represented by *trans*-NATs in which the expression between lncRNAs and coding genes do not vary; the central group is composed of *trans*-NATs in which the coding genes are overexpressed compared to paired lncRNAs; and the right-side group is composed of *trans*-NATs in which the lncRNAs are overexpressed compared to paired coding genes. Interestingly, these *trans*-NATs are pairing coding genes enriched (*p* < 0.05) on the ‘molecular function’ GO categories ‘Nicotianamine synthase activity’, ‘transcription factor activity’ and ‘transcription regulator activity’ (Table S6). These pairs are also enriched in ‘biological process’ GO categories normally associated with response to stress and abiotic stimulus. Figure 8B presents the top enriched ‘biological process’ GO categories. These results suggest that lncRNAs probably play important roles on the regulation of key protein coding genes associated with stress tolerance in Lluteño maize. The lists of all GO enriched categories is made available (Table S6).

## 4. Discussion

Increasing evidence has demonstrated that plant lncRNAs play important roles in multiple biological processes, such as developmental regulations and stress responses [7,10,18,31,65,72]. The majority of well-studied lncRNAs are from animals, while the biological functions of plant lncRNAs remain poorly characterized. Here, we conducted a genome-wide systematic identification of lncRNAs that responds to a combination of salinity and boron stress in Lluteño maize, a local landrace from Atacama Desert adapted to soil with high concentration of these elements [35]. Salinity and excess of boron are two common environmental stresses that usually act together in some arid and semi-arid regions worldwide, such as South Australia, Israel, San Joaquin Valley in California and Lluta Valley in north of Chile [73].

We identified 48,345 lncRNAs transcripts in Lluteño maize, and 26,639 of them were found to be present in the reference B73 maize genome assembly (55.1%). When comparing Lluteño lncRNAs with other non-reference maize genomes (Mo17 and Palomero), we found that over 41.9% had no significant matches by sequence similarity searches with publicly available genomes, suggesting that the repertoire and complexity of maize non-coding transcriptional activity is still poorly studied. This presumable incidence of Lluteño exclusive transcripts may suggests a rapid evolution of maize lncRNAs, or that during its evolution under a drastic stress environment, induced by the combination of salinity and excess of boron, Lluteño maize had developed a set of specifics lncRNAs to regulate the gene expression and its cellular activity. In animals, lncRNAs are known to evolve rapidly and to be poorly conserved [74]. The pipeline used here for the identification of maize lncRNAs is similar to that used in other studies in plants [17,72,75,76,77]. In this approach, we did not use a strand-specific library, thus complicating the identification of the transcriptional orientation of these transcripts. However, despite this, a relatively robust and reliable list of maize lncRNAs is provided. The bioinformatics characterization performed and expression validation by qPCR gives supports that these transcripts might be bona fide lncRNAs.

Secondary structures are important for stabilizing RNA molecules and can have key roles on their functional activity in cellular processes [67,78]. We found that Lluteño lncRNAs presents more significant low energies compared to random sets of sequences. This suggests that there is an evolutionary driving force that maintains this thermodynamic low free energy compared to alternative folds of random sequences. We also investigated if these long ncRNAs could be precursors of small RNAs in Lluteño maize. Small RNAs are important classes of ncRNAs for the regulation of gene expression in plants, which can be originated from longer RNA precursors processed by Dicer-like 1 [79]. Recently, 10 out of 410 gibberellin-responsive lncRNAs as precursors of 17 known miRNAs in *Populus* were identified [72]. From the complete dataset of Lluteño lncRNAs, we identified 170 potential miRNAs precursors, which are conserved among other plants species based on sequence similarity searches according to the National Radiology Data Registry (NRDR) database [4]. The target transcripts of these miRNAs should be further investigated to verify if they are regulators of protein coding genes or other lncRNAs.

We analyzed the genomic context of conserved lncRNAs according to B73 reference genome sequence and classified them based on their genomic relationship with protein coding genes as intergenic or intragenic (overlapping exonic or intronic regions). We found that nearly 75% of conserved lncRNAs (17,550 out of 22,857) were long intergenic non-coding RNAs (lincRNAs), similar to the results reported to tomato, in which over 80% of all lncRNAs identified in wild-type tomato and mutant ripening fruit came from intergenic regions [10]. In a comprehensive study in human, Iyer and collaborators [80] identified many lncRNAs associated (about 59,000) to normal, tumors tissues and cell lines from 25 independent studies. Similar to our study, a high percentage of these human lncRNAs (79%, 47,000) were unannotated relative to databases and most (72%) were located within intergenic regions. Co-expression analysis based on Pearson correlation revealed that part of Lluteño lncRNAs could be regulating, positively or negatively, their neighbor genes. It is widely known that lncRNAs may act regulating the expression of neighbor genes, by recruiting protein complexes that modifies chromatin architecture [81,82]. The role of these lncRNAs should be further characterized to identify a directly regulation of genes associated with stress tolerance in maize.

Long non-coding RNAs can also regulate other transcripts in *trans*, i.e., transcribed from different loci [17,83]. For instance, *trans*-NAT are defined as RNA molecules originated from a particular region of the genome, that regulate other transcripts generated from different loci by base pairing complementary regions [12], at the transcriptional and/or post-transcriptional level [84]. We identified 32,335 *trans*-NAT pairs in Lluteño maize, with the majority of lncRNAs pairing to coding genes (85.89% of all pairs). Interestingly, target genes were enriched in GO categories associated to gene regulation (i.e., ‘sequence-especific DNA binding’ and ‘transcription regulator activity’) and to stress response (i.e., ‘response to abiotic stimulus’ and ‘response to homeostasis’). We also found a set of lncRNAs associated with protein coding genes involved in nicotianamine biosynthetic and metabolic process. Recently, *Arabidopsis* transgenic lines overexpressing the Nicotianime synthase gene (*TaNAS-D*) from wheat showed an enhanced salt stress tolerance [85]. The role of this particularly *trans*-NAT subgroup should be further studied.

Numerous lncRNAs associated to abiotic stress have been identified in several species, such as *Arabidopsis* [12,18,32], rice [76], maize [33,77], wheat [15], *Setaria* [86] and *Populus* [65,75,87]. Here, we found that a set of lncRNAs possess higher expression levels than coding genes in tissues more exposed to stress conditions, like roots under 96 h of exposure. This higher expression levels differs from the one reported previously for lncRNAs [88], in which coding genes normally possess higher levels of expression. These expression levels of lncRNAs could be an adaptability of the plant to surpass the stressed environment. In this study, we identified 1710 lncRNAs statistically differentially expressed under a combined condition of salinity and boron stress, including 955 conserved and 755 Lluteño exclusive lncRNAs in comparison to other maize variants. The dynamic changes of lncRNAs expression during stress conditions indicated that lncRNAs might play significant biological roles in response to a combined salinity and boron stress. Salt and boron responsive lncRNAs identified in this study were selected by bioinformatics analysis and confirmed experimentally. Our results showed that several lncRNAs exhibited leaves or roots specifics expression patterns. Twelve were further demonstrated experimentally by RT-qPCR, showing significant changes in their activity in leaves or roots stressed by salinity and boron, in accordance with our RNA-seq analysis.

Altogether, these results are in accordance with the vastness and complexity of lncRNA transcription, which has been grossly underappreciated [80], and particularly suggest that there are a set of lncRNAs in Lluteño maize that could also play an important role at a post-transcriptional regulation level, in the adaptation and stress response to soil with high concentration of salinity and boron. Although the biological function of the most lncRNAs is unknown, several approaches could provide a useful way to determine their biological functions, such as the prediction and analysis of target coding genes, interactions with microRNAs, co-expression analysis together with coding genes, silencing and overexpression of particular lncRNAs, etc. Our findings constitute a valuable resource of stress responsive lncRNAs for future research in this direction. This is first report focused on identifying lncRNAs associated to a combined stress induced by high salinity and boron, using a maize landrace from Atacama Desert, adapted to environments with high concentration of these elements.

## Figures and Tables

**Table 1:** *Trans* natural transcript *(trans*-NAT) pairs classification and expression distribution in all conditions.

Sample	100-nt Class				High-Coverage Class			
	Both	None	Only Coding	Only lncRNA	Both	None	Only Coding	Only lncRNA
**Leaf Control**	3532	1124	952	2742	5781	5095	3777	4770
**Leaf 3 h**	3109	1305	1062	2874	5024	5789	4200	4410
**Leaf 96 h**	3950	8130	787	2800	6892	3878	3386	5267
**Root Control**	3026	1328	1312	2684	5775	4968	4104	4576
**Root 3 h**	2891	1444	1235	2780	5812	4825	3894	4892
**Root 96 h**	2380	2209	1428	2333	4081	7238	4625	3479

## Supplementary Materials

The following are available online at http://www.mdpi.com/2073-4425/9/3/170/s1, File S1: FASTA file containing all identified Lluteño lncRNAs. Figure S1: Curve of reads used to reconstruct the total number of transcripts. Random reads subsamples were used to count the number of covered transcripts in intervals composed by the increment of one million reads until it reaches the total number of high quality reads. Figure S2: Histogram of *z-*score frequencies for all studied lncRNAs based on AlifoldZ approach [68]. All z-scores smaller than −5 were approximated to −5. Figure S3: Lluteño maize expressed lncRNAs in control, 3 h and 96 h of exposure to salt and boron. Table S1: List of primers used on the quantitative PCR validation. Table S2: Chromosomal distribution of Lluteño lncRNAs according to B73 genome sequence and protein coding genes. Table S3: Excel table of the Pearson correlations between the expression levels of Lluteño lncRNAs and the closest coding genes according to B73 genome. Table S4: Excel table of differentially expressed lncRNAs. The table contains the expression values, fold-changes for each condition and its presence in other maize genomes (B73, Palomero and MO17). Table S5: Gene Ontology enriched categories of lncRNA-coding gene *trans*-NAT pairs. P = Biological Process, C = Cellular Component, F = Molecular Function. Table S6: Gene Ontology enriched categories of lncRNA-coding gene *trans*-NAT pairs. P = Biological Process, C = Cellular Component, F = Molecular Function.
