# Supplementary material for: Long Non-Coding RNAs Responsive to Salt and Boron Stress in the Hyper-Arid Lluteño Maize from Atacama Desert
Source: Genes (Basel). 2018 Mar 20;9(3):170. doi: 10.3390/genes9030170 (PMC5867891; doi:10.3390/genes9030170)
Supplement: Supplementary file 1 [file genes-09-00170-s001.zip › Suplementary_Table_1.docx]

Supplementary Table 1. List of primers used on the quantitative PCR validation. The transcript ID, primer sequences and product size are represented.

| ID | Primer sequence (5'---> 3') | | Product size (bp) |
| --- | --- | --- | --- |
|  | Forward | Reverse |  |
| LZM13258 | AGTGGCGATGGAATTGAGTG | CCTCCCCTGTAACAAACTTTCG | 145 |
| LZM00136 | GCGGCACGTACTCTATTCTG | TTGACCTGTGTTCTGAGTTCTG | 138 |
| LZM48210 | CCTAGGAGTGGAAATTAAACGACC | GGTGAAATTGATCTGCGTGTG | 167 |
| LZM15422 | GTACGTAAGGATGTGCTGAGG | CTCCACATGATCGATAGCCAG | 100 |
| LZM16003 | TGCGTCTCCGATGTTTGAAG | GGAGCACATCACGATCTTAGTC | 135 |
| LZM12230 | CATGGTTTTGGGTGCTGATG | TCCGATCCCACAGAAACTTTT | 87 |
| LZM16074 | CCGTGTCTGTGTCGTGTATTC | TGGATGAAATGGAGTCAGCTC | 149 |
| LZM04165 | GAGATTTGGGAGAGTGGAGTTC | AGGCTAAATTCAGATGAGGTGG | 156 |
| LZM14981 | ATTATCCTGTATGCTCCGTGC | GTCCAGTCAAGAAAGGCGG | 160 |
| LZM02126 | CCAATAAGGCGAAGCTCCAG | CAGGAGCAGGGAAAGGAC | 139 |
| LZM15656 | CAGCTACTAGGACACAAGACAG | CGATCGCTCCAGAAATGAACG | 84 |
| LZM14857 | GAAAGCCTGAAACTGCTCAAG | GTTGGGAGCTAGAGGAAAGAG | 89 |
