# Supplementary material for: Long Non-Coding RNAs Responsive to Salt and Boron Stress in the Hyper-Arid Lluteño Maize from Atacama Desert
Source: Genes (Basel). 2018 Mar 20;9(3):170. doi: 10.3390/genes9030170 (PMC5867891; doi:10.3390/genes9030170)
Supplement: Supplementary file 1 [file genes-09-00170-s001.zip › Supplementary_Material.docx]

Supplementary Material

Long non-coding RNAs responsive to salt and boron stress in the hyper-arid lluteño maize from Atacama Desert

**Wilson Huanca-Mamani^*^, Raul Arias-Carrasco, Steffany Cárdenas-Ninasivincha, Marcelo Rojas-Herrera, Gonzalo Sepúlveda-Hermosilla, José Carlos Caris-Maldonado, Elizabeth Bastías and Vinicius Maracaja-Coutinho^*^**

*** Correspondence:** Corresponding Authors: whuanca@uta.cl and viniciusmaracaja@integrativebioinformatics.me

# Supplementary Data

**Supplementary Data 1.** Fasta file containing all identified Lluteño lncRNAs.

# Supplementary Figures and Tables

## Supplementary Figures

##
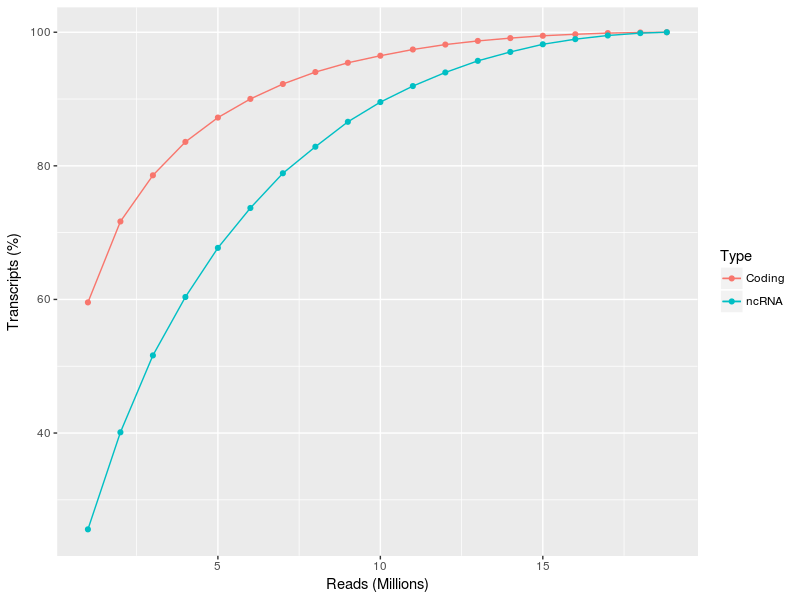


## Supplementary Figure 1. Curve of reads used to reconstruct the total number of transcripts. Random reads subsamples were used to count the number of covered transcripts in intervals composed by the increment of one million reads until it reaches the total number of high quality reads.


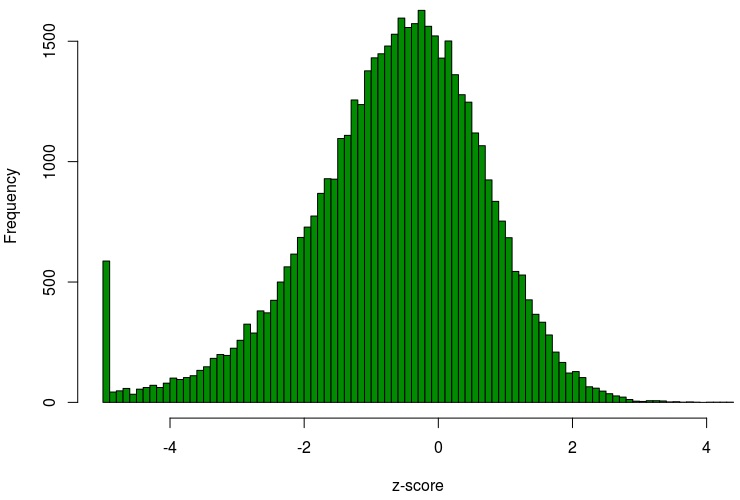


**Supplementary Figure 2.** Histogram of z-score frequencies for all studied lncRNAs based on AlifoldZ approach (Washielt and Hofacker, 2004). All z-scores smaller than -5 were approximated to -5.

**
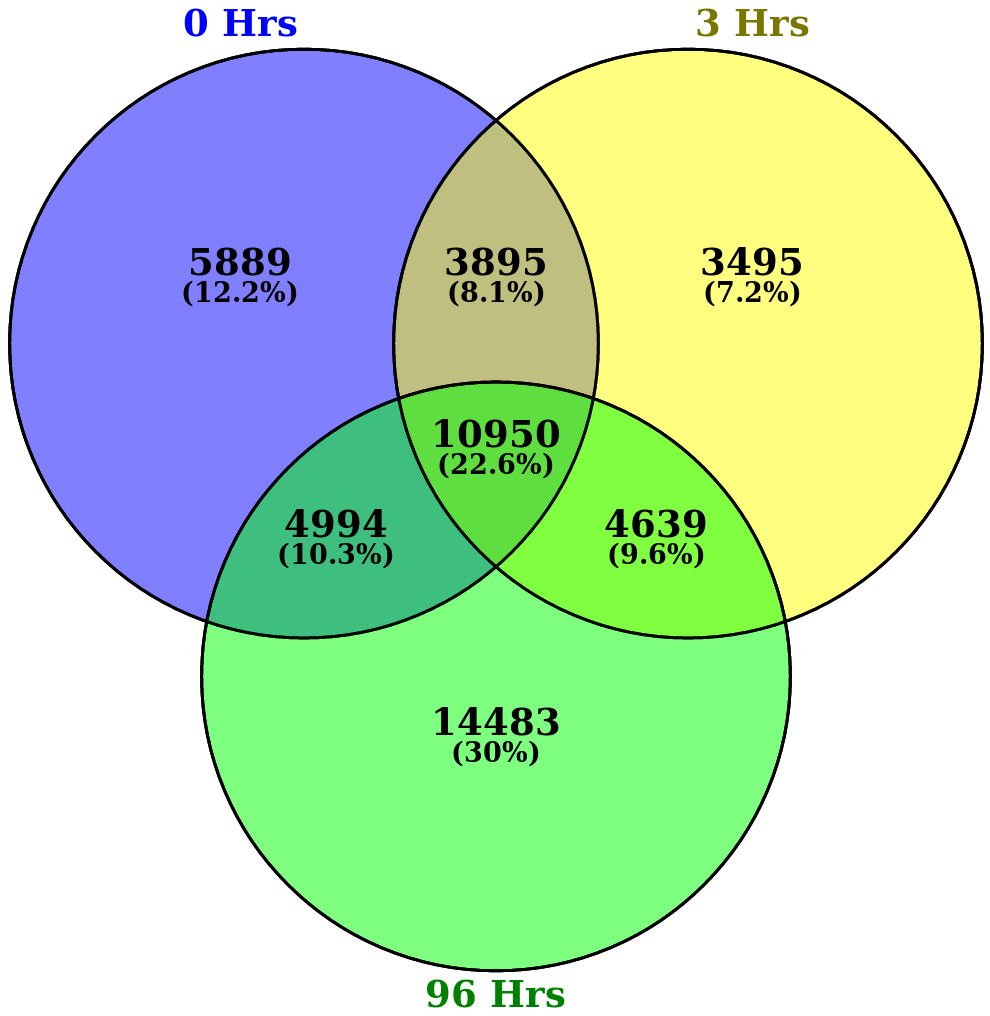
**

**Supplementary Figure 3.** Lluteño maize expressed lncRNAs in control, 3 hours and 96 hours of exposure to salt and boron.

## Supplementary Tables

**Supplementary Table 1.** List of primers used on the quantitative PCR validation. Transcript ID, primers sequences and product size.

**Supplementary Table 2.** Chromosomal distribution of Lluteño lncRNAs according to B73 genome sequence and protein coding genes.

**Supplementary Table 3.** Excel table of the Pearson correlations between the expression levels of Lluteño lncRNAs and the closest coding genes according to B73 genome

**Supplementary Table 4.** Excel table of differentially expressed lncRNAs. The table contains the expression values, fold-changes for each condition and its presence in other maize genomes (B73, Palomero and MO17).

**Supplementary Table 5.** Gene Ontology enriched categories of lncRNA-coding gene *trans*-NAT pairs. P = Biological Process, C = Cellular Component, F = Molecular Function.

**Supplementary Table 6.** Gene Ontology enriched categories of lncRNA-coding gene *trans*-NAT pairs. P = Biological Process, C = Cellular Component, F = Molecular Function.

**References**

Washietl, S., and Hofacker, I. L. (2004). Consensus folding of aligned sequences as a new measure for the detection of functional RNAs by comparative genomics. *J. Mol. Biol.* 342, 19–30.
