# Supplementary figures and images for: Long Non-Coding RNAs Responsive to Salt and Boron Stress in the Hyper-Arid Lluteño Maize from Atacama Desert
Source: Genes (Basel). 2018 Mar 20;9(3):170. doi: 10.3390/genes9030170 (PMC5867891; doi:10.3390/genes9030170)

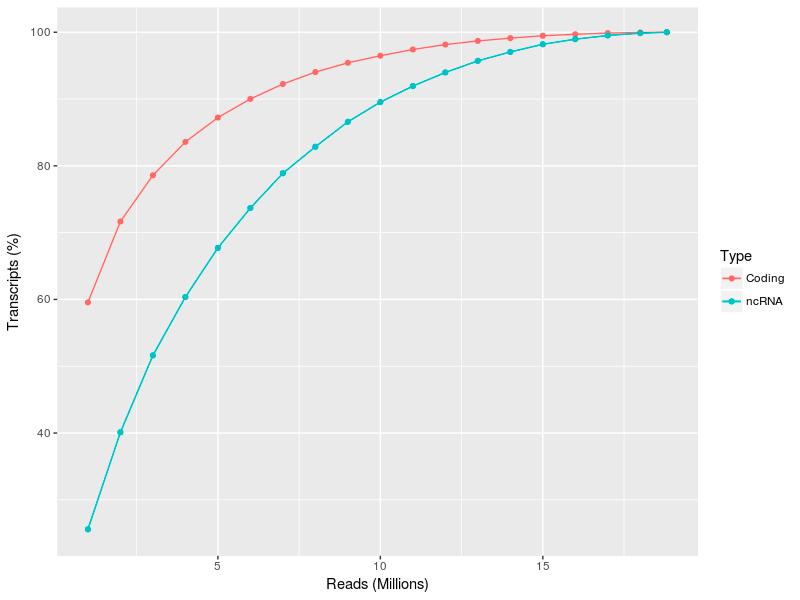

Supplement: Supplementary file 1 [file genes-09-00170-s001.zip › Supplementary_Figure_1.jpeg]

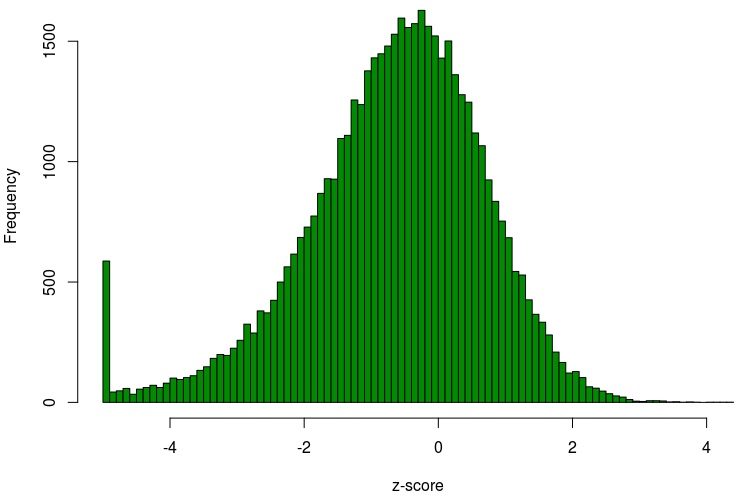

Supplement: Supplementary file 1 [file genes-09-00170-s001.zip › Supplementary_Figure_2.jpeg]

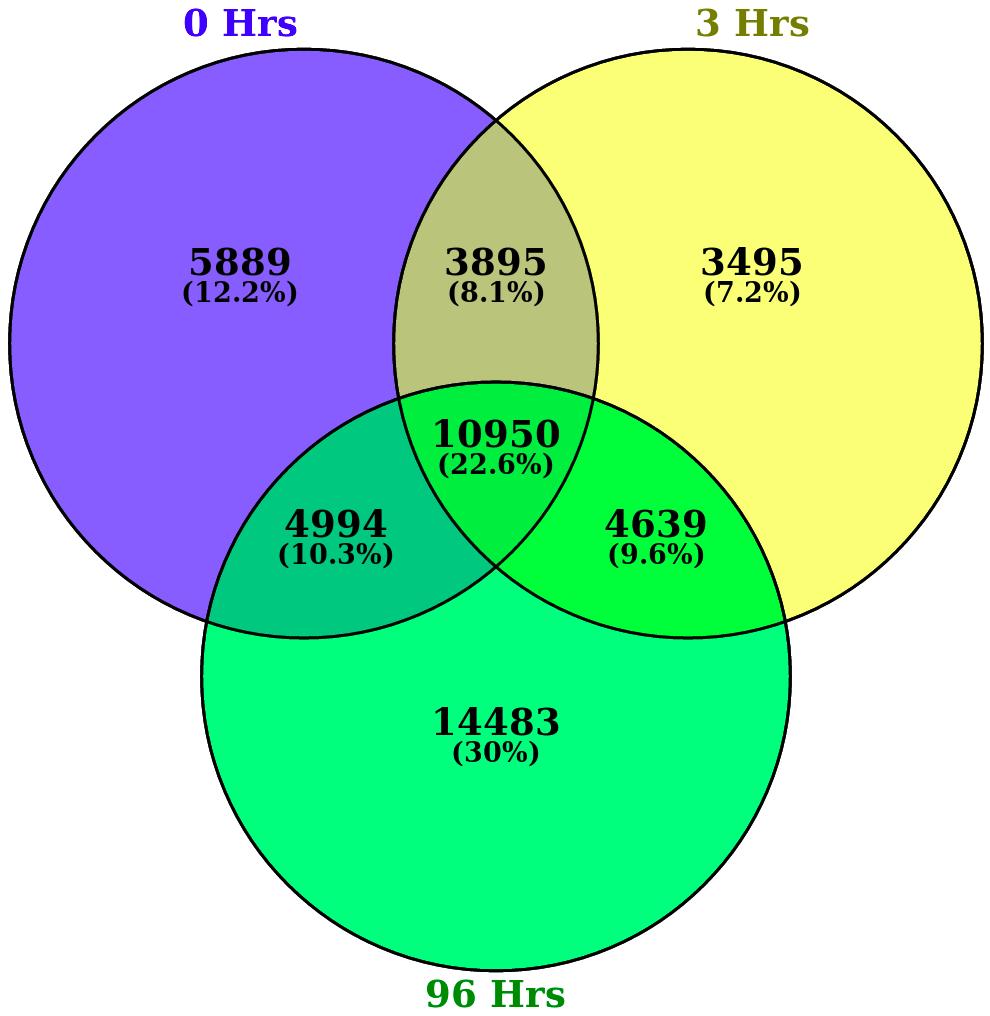

Supplement: Supplementary file 1 [file genes-09-00170-s001.zip › Supplementary_Figure_3.jpeg]
